# Supplementary material for: Producing polished prokaryotic pangenomes with the Panaroo pipeline
Source: Genome Biol. 2020 Jul 22;21:180. doi: 10.1186/s13059-020-02090-4 (PMC7376924; doi:10.1186/s13059-020-02090-4)
Supplement: Supplementary file 1 — Additional file 1 Additional file 1 includes all supplementary tables and figures. [file 13059_2020_2090_MOESM1_ESM.pdf]

Additional file 1

Gain/Loss Ratio	Mutation Rate	Source of Error
0.1	1.00E-14	NA
1	1.00E-14	NA
10	1.00E-14	NA
10	1.00E-15	NA
10	1.00E-16	NA
10	1.00E-14	Fragmentation
10	1.00E-14	Contamination with *S. epidermidis*

Table S1: Parameters used to simulate pangenomes using the Infinitely Many Genes model.

Gene	lrt-pvalue	beta	beta-std-err	Antibiotic	Annotation
gatC	9.38E-06	0.402	0.0903	PEN	aspartyl/glutamyl-tRNA amidotransferase subunit C
group_111	4.82E-07	0.319	0.063	PEN	ATP synthase F0F1 subunit delta
group_1126	3.60E-08	0.18	0.0324	PEN	putative thiosulfate sulfurtransferase
group_1140	9.18E-06	-0.108	0.0242	AZM	ArsR family transcriptional regulator
group_1168	2.95E-10	0.317	0.0498	PEN	integral membrane protein
group_1237	1.92E-20	0.165	0.0174	TET	putative phage associated protein;phage associated protein
group_1333	4.44E-07	0.263	0.0517	CFM	two-component system transcriptional response regulator
group_1333	5.93E-18	0.499	0.0568	PEN	two-component system transcriptional response regulator
group_1333	4.44E-07	0.263	0.0517	CRO	two-component system transcriptional response regulator
group_134	4.48E-06	0.211	0.0458	CRO	TonB-dependent receptor protein
group_134	2.30E-19	0.439	0.0478	PEN	TonB-dependent receptor protein
group_134	4.48E-06	0.211	0.0458	CFM	TonB-dependent receptor protein
group_1387	5.01E-09	0.12	0.0203	TET	putative maltose phosphorylase
group_144	9.10E-06	0.149	0.0334	PEN	elongation factor G
group_1491	3.64E-08	0.261	0.047	PEN	phage protein
group_1496	2.09E-08	0.332	0.0587	PEN	membrane protein
group_1511	2.17E-07	0.377	0.0722	PEN	phage protein
group_1611	9.18E-06	-0.108	0.0242	AZM	phage associated protein;hypothetical protein
group_1623	2.65E-07	0.256	0.0494	CRO	phage associated protein
group_1623	1.34E-17	0.486	0.0559	PEN	phage associated protein
group_1623	2.65E-07	0.256	0.0494	CFM	phage associated protein
group_1693	8.09E-15	0.122	0.0155	TET	putative cytochrome C
group_1708	2.65E-07	0.256	0.0494	CFM	VapD-like protein
group_1708	1.34E-17	0.486	0.0559	PEN	VapD-like protein
group_1708	2.65E-07	0.256	0.0494	CRO	VapD-like protein
group_172	7.24E-06	0.202	0.0447	PEN	IS1016 transposase
group_1753	2.42E-08	0.419	0.0745	CRO	YegA
group_1753	4.11E-09	0.472	0.0796	PEN	YegA
group_1753	2.42E-08	0.419	0.0745	CFM	YegA
group_238	1.78E-08	-0.0668	0.0118	TET	amidophosphoribosyltransferase
group_299	4.82E-07	0.319	0.063	PEN	NADH:ubiquinone dehydrogenase L subunit
group_380	9.10E-06	0.149	0.0334	PEN	arsenate reductase
group_438	9.18E-06	-0.108	0.0242	AZM	phosphoribosylaminoimidazole carboxylase ATPase subunit
group_451	2.42E-08	0.419	0.0745	CRO	Protein rnfH
group_451	4.11E-09	0.472	0.0796	PEN	Protein rnfH
group_451	2.42E-08	0.419	0.0745	CFM	Protein rnfH
group_464	2.22E-08	0.178	0.0316	PEN	ABC transporter ATP-binding protein
group_914	9.62E-07	0.0566	0.0115	CIP	phage repressor phage associated protein
group_945	4.82E-07	0.319	0.063	PEN	ABC transporter permease amino acid
porB	1.63E-06	-0.152	0.0316	PEN	major outer membrane protein porin P.IB; P.I
rplW	1.53E-08	-0.327	0.0574	AZM	50S ribosomal protein L23

Table S2: Significant pan-GWAS results for antibiotic resistance in the EuroGASP collection

variant	af	filter-pvalue	lrt-pvalue	beta	beta-std-err	variant_h2	antibiotic
metG-group_1858-pilE_1_pilE_pilE_3_pilS5_pilS2_1_pilE_2	0.0398	1.79E-09	4.85E-45	-0.36	0.0244	0.415	CIP
group_946-group_1499-group_1846	0.0133	0.0379	5.08E-45	0.317	0.0215	0.415	TET
hemC-group_1846-group_1499	0.0133	0.0379	5.08E-45	0.317	0.0215	0.415	TET
group_1056-group_1846-pepN	0.0218	7.10E-35	3.75E-43	-0.377	0.0261	0.406	TET
group_885-group_1826-group_1827	0.0275	1.13E-45	7.11E-43	-0.273	0.019	0.405	TET
group_143-group_933-group_1118	0.0133	2.26E-10	2.84E-39	0.31	0.0226	0.388	TET
group_212-group_592-group_1675	0.0199	2.62E-31	1.90E-31	-0.256	0.0212	0.348	TET
group_644-group_801-group_505	0.0256	4.65E-42	5.58E-29	-0.257	0.0223	0.335	TET
group_1236-group_1556-group_1237	0.0598	8.52E-07	1.83E-28	0.23	0.0202	0.332	TET
group_505-group_1775-group_1232	0.0247	2.95E-40	4.42E-27	-0.251	0.0227	0.323	TET
group_1775-group_505-group_801	0.0237	1.85E-38	1.11E-23	-0.231	0.0225	0.302	TET
group_238-group_1556-group_1236	0.0949	3.87E-42	1.83E-22	-0.189	0.019	0.294	TET
group_936-group_885-group_1826	0.102	1.26E-19	3.17E-22	-0.121	0.0122	0.292	TET
group_1367-group_1237-group_1556	0.0294	0.76	1.11E-21	0.206	0.0211	0.289	TET
group_1824-group_164-group_1899	0.0123	0.0199	1.53E-20	0.176	0.0185	0.281	CIP
group_1229-group_1118-group_933	0.0218	6.11E-25	3.74E-20	0.183	0.0195	0.278	TET
group_936-group_885-group_1827	0.0417	0.971	6.45E-19	0.123	0.0136	0.269	TET
group_1335-group_1794-group_1099	0.018	0.00505	2.90E-18	0.358	0.0403	0.264	CRO
group_1720-group_75-group_1901	0.0503	7.80E-79	1.96E-17	-0.258	0.0299	0.258	SMX
group_1367-group_238-group_1556	0.102	4.08E-50	1.51E-16	-0.142	0.0169	0.251	TET
group_1393-group_1795-group_1690	0.0104	0.00778	4.41E-16	0.204	0.0247	0.247	TET
group_1032-group_1894-group_1051	0.0123	0.226	7.84E-16	0.145	0.0177	0.245	CIP
group_1630-ppsR-group_1489	0.0237	0.203	1.78E-15	-0.147	0.0182	0.242	CIP
ppsA-group_1489-ppsR	0.0237	0.203	1.78E-15	-0.147	0.0182	0.242	CIP
group_1332-group_1826-group_117	0.0114	0.0123	7.75E-15	-0.163	0.0206	0.236	CIP
rho-ppsA-group_1489	0.0247	0.145	9.58E-15	-0.14	0.0178	0.235	CIP
group_136-group_1367-group_1237	0.037	0.398	1.12E-14	0.136	0.0174	0.235	TET
group_68-group_64-group_37	0.0342	0.000752	1.25E-14	-0.126	0.0161	0.234	CIP
group_64-group_37-group_915	0.0342	0.000752	1.25E-14	-0.126	0.0161	0.234	CIP
group_1333-group_117-group_1826	0.0123	0.00691	1.92E-14	-0.157	0.0202	0.233	CIP
group_190-group_577-group_150	0.0294	0.377	2.73E-14	0.111	0.0144	0.231	TET
hemC-group_1846-pepN	0.0266	3.68E-17	6.23E-14	-0.141	0.0186	0.228	TET
rpsS-rplB-group_1157	0.829	7.48E-31	6.85E-14	0.575	0.0757	0.228	AZM
rpsS-rplB-rplW	0.171	7.48E-31	6.85E-14	-0.575	0.0757	0.228	AZM
group_676-mafB_mafB_2-group_1127	0.0266	0.000891	1.19E-13	0.675	0.0898	0.226	CRO
group_1895-group_493-group_1591	0.858	2.81E-35	1.91E-13	0.56	0.0752	0.224	AZM
group_702-group_1633-group_1869	0.0171	0.458	1.93E-13	0.142	0.019	0.224	TET
group_1051-group_460-group_1482	0.0104	0.0217	2.24E-13	0.128	0.0172	0.223	CIP
group_446-pepN-group_1846	0.0531	1.40E-10	1.20E-12	-0.107	0.0149	0.217	TET
group_972-group_1612-group_1829	0.0323	8.11E-07	1.36E-12	0.104	0.0145	0.216	CIP
group_563-group_303-group_1171	0.0218	9.75E-17	1.79E-12	-0.138	0.0193	0.215	TET
group_2008-group_1991-group_1972	0.135	1.55E-05	2.46E-12	-0.105	0.0148	0.214	TET
group_393-group_262-group_1171	0.0199	3.84E-16	4.36E-12	-0.141	0.0201	0.211	TET
group_303-group_1171-group_262	0.019	1.31E-14	6.45E-12	-0.142	0.0204	0.209	TET
group_1121-group_1531-group_1176	0.0228	0.458	8.01E-12	0.202	0.0292	0.209	AZM
group_255-group_841-group_1576	0.488	4.57E-82	8.19E-12	0.312	0.0451	0.208	PEN
metG-group_1576-group_841	0.488	4.57E-82	8.19E-12	0.312	0.0451	0.208	PEN
group_758-group_841-group_255	0.512	4.57E-82	8.19E-12	-0.312	0.0451	0.208	PEN
group_758-group_1576-metG	0.512	4.57E-82	8.19E-12	-0.312	0.0451	0.208	PEN
group_2024-group_1993-group_1985	0.14	1.43E-05	1.03E-11	-0.0985	0.0143	0.207	TET
rho-ppsA-ppsR	0.0285	0.00396	3.69E-11	0.0904	0.0135	0.202	CIP
group_1630-ppsR-ppsA	0.0285	0.00396	3.69E-11	0.0904	0.0135	0.202	CIP
dut-group_385-group_1465	0.0104	0.0225	4.32E-11	0.603	0.0906	0.201	CRO
group_1066-group_1531-group_1176	0.544	1.83E-09	4.49E-11	-0.173	0.026	0.201	AZM
group_1711-group_1694-group_1894	0.0266	1.94E-27	8.25E-11	-0.135	0.0205	0.198	TET
pyrG-group_1870-group_714	0.0304	0.74	1.07E-10	0.235	0.036	0.197	PEN
group_95-group_363-group_1224	0.0455	3.36E-10	1.12E-10	-0.311	0.0478	0.197	CIP
group_363-group_95-group_1299	0.0455	3.36E-10	1.12E-10	-0.311	0.0478	0.197	CIP
group_177-group_791-group_892	0.0152	7.03E-07	3.50E-10	-0.13	0.0205	0.192	TET
group_1066-group_1121-group_1558	0.97	0.327	4.70E-10	-0.162	0.0257	0.19	AZM
group_182-group_714-group_1870	0.0114	1.79E-12	8.68E-10	-0.154	0.0249	0.187	TET
group_1134-alaS-group_67	0.0304	0.00204	1.14E-09	0.0918	0.0149	0.186	TET
group_283-group_67-alaS	0.0304	0.00204	1.14E-09	0.0918	0.0149	0.186	TET
group_185-group_274-group_908	0.737	1.49E-08	1.42E-09	-0.0695	0.0114	0.185	CIP
group_2007-group_1985-group_1993	0.214	0.175	1.58E-09	-0.0818	0.0134	0.185	TET
group_1531-group_1121-group_1558	0.0275	0.637	1.60E-09	0.157	0.0258	0.184	AZM
group_1401-group_1332-group_1826	0.0161	0.343	1.92E-09	-0.0982	0.0162	0.184	CIP
group_1824-group_164-opaD_piiC	0.0332	0.0109	2.68E-09	-0.0883	0.0147	0.182	CIP
group_292-group_412-group_321	0.0342	2.07E-07	4.03E-09	0.143	0.024	0.18	AZM
group_1126-mafB_mafB_2-group_1136	0.315	1.17E-10	4.05E-09	0.205	0.0345	0.18	PEN
group_2026-group_2011-group_2005	0.225	0.201	7.69E-09	-0.0756	0.013	0.177	TET
group_1066-group_1531-group_1190	0.426	1.44E-11	7.80E-09	-0.183	0.0314	0.177	CRO
group_683-group_385-group_1738	0.0503	0.0135	8.26E-09	0.473	0.0815	0.176	CRO
group_1516-group_1056-group_1846	0.0152	1.77E-22	9.22E-09	-0.149	0.0257	0.176	TET
group_702-group_1633-group_1902	0.0209	0.00462	9.51E-09	0.0917	0.0158	0.176	CIP
ispH-lspA-group_1875	0.0588	0.000396	1.14E-08	0.106	0.0184	0.175	AZM
rplB-group_1157-rplD	0.824	1.14E-30	1.53E-08	0.327	0.0574	0.173	AZM
rplC-rplD-group_1157	0.824	1.14E-30	1.53E-08	0.327	0.0574	0.173	AZM
rplC-rplD-rplW	0.176	1.14E-30	1.53E-08	-0.327	0.0574	0.173	AZM
group_976-group_494-group_1552	0.278	7.66E-09	1.65E-08	-0.109	0.0192	0.173	AZM
group_494-group_1552-tbpA	0.278	7.66E-09	1.65E-08	-0.109	0.0192	0.173	AZM
pilS1_pilE_pilE_1_pilE1_pilS6_2_pilS5_pilS2_pilS6_pilS6_1_pilE_2_pilE_3-pilE_1_pilS6_pilE_pilE1-pilE_pilE_1_pilS6_pilE1_pilE_2	0.0408	0.432	2.87E-08	0.178	0.0317	0.17	CRO
pilS5_pilE_pilS2-pilE_1_pilE1_pilS6-pilS6_pilE_pilS2	0.0104	7.70E-11	3.06E-08	0.161	0.0288	0.17	TET
group_205-pyrG-group_1870	0.0361	0.734	3.16E-08	0.191	0.0343	0.169	PEN
group_903-group_1759-group_1716	0.0142	0.0115	3.39E-08	-0.177	0.0319	0.169	CRO
group_1404-group_321-group_412	0.038	1.37E-07	3.51E-08	0.126	0.0226	0.169	AZM
group_1751-group_1224-group_363	0.0465	1.84E-10	4.12E-08	-0.226	0.041	0.168	CIP
group_1951-group_46-group_886	0.0123	0.597	4.21E-08	-0.351	0.0636	0.168	PEN
group_451-group_714-group_1870	0.0218	0.136	4.52E-08	0.246	0.0446	0.167	PEN
group_1761-group_1801-group_1749	0.0664	6.69E-05	5.18E-08	-0.0652	0.0119	0.167	TET
group_1103-group_953-group_899	0.0152	0.00122	6.37E-08	0.16	0.0294	0.166	CIP
group_185-group_274-group_167	0.0408	9.82E-10	7.58E-08	0.0633	0.0117	0.165	CIP
group_536-group_940-group_252	0.0199	1.41E-21	8.45E-08	0.162	0.03	0.164	TET
group_940-group_252-group_757	0.0199	1.41E-21	8.45E-08	0.162	0.03	0.164	TET
group_1514-group_679-porB	0.229	5.98E-14	8.51E-08	-0.16	0.0296	0.164	PEN
lpxC-pilS5_pilE_2_pilS5_2_pilE_pilE1_pilE_1_pilE_4-group_1778	0.0133	0.00407	8.87E-08	0.133	0.0247	0.164	CRO
group_136-group_1367-group_238	0.166	5.32E-37	1.11E-07	-0.0617	0.0115	0.163	TET
ileS-group_1875-lspA	0.0408	0.0324	1.13E-07	0.116	0.0217	0.162	AZM
group_1126-mafB_mafB_2-group_1100	0.766	4.67E-24	1.21E-07	0.171	0.0321	0.162	PEN
group_976-group_494-group_1864	0.0787	5.06E-15	1.25E-07	0.101	0.019	0.162	AZM
group_1136-mafB_mafB_2-group_1127	0.0607	0.191	1.30E-07	0.247	0.0465	0.162	PEN
group_1265-group_1890-group_1901	0.0209	3.42E-06	1.39E-07	-0.367	0.0691	0.161	PEN
pip-opaD_piiC-group_164	0.0275	0.11	1.54E-07	-0.0839	0.0159	0.161	CIP
group_992-group_128-group_1602	0.018	3.33E-21	2.32E-07	0.158	0.0304	0.158	TET
group_1562-group_1190-group_1176	0.319	1.63E-09	2.53E-07	0.109	0.021	0.158	SMX
lpxC-pilS5_pilE_2_pilS5_2_pilE_pilE1_pilE_1_pilE_4-pilS5_pilE_pilS2	0.0142	0.0115	2.57E-07	0.123	0.0238	0.158	CRO
group_861-group_485-group_935	0.0266	3.94E-05	2.91E-07	0.0586	0.0113	0.157	CIP
group_1541-group_68-group_914	0.108	2.47E-14	3.67E-07	-0.0443	0.00866	0.156	TET
group_68-group_914-group_37	0.108	2.47E-14	3.67E-07	-0.0443	0.00866	0.156	TET
group_1374-pilE1_pilE_1_pilE__pilE_2_pilS3_pilS6-pilS5_pilE_2_pilS5_2_pilE_pilE1_pilE_1_pilE_4	0.0427	0.000119	4.00E-07	0.105	0.0206	0.155	TET
group_914-group_37-group_915	0.694	5.88E-10	4.11E-07	0.0588	0.0115	0.155	CIP
group_495-ileS-group_1875	0.0636	0.000141	4.79E-07	0.104	0.0205	0.154	AZM
group_1613-group_504-group_412	0.0114	0.0924	5.28E-07	-0.259	0.0513	0.154	AZM

variant	af	filter-pvalue	lrt-pvalue	beta	beta-std-err	variant_h2	antibiotic
group_303-group_1450-group_262	0.0142	1.14E-05	6.64E-07	-0.113	0.0226	0.152	TET
group_1131-group_795-group_1138	0.743	0.123	6.87E-07	0.322	0.0645	0.152	CRO
group_771-group_1002-group_1138	0.743	0.123	6.87E-07	0.322	0.0645	0.152	CRO
group_1002-group_795-group_1131	0.257	0.123	6.87E-07	-0.322	0.0645	0.152	CRO
group_771-group_1002-group_795	0.257	0.123	6.87E-07	-0.322	0.0645	0.152	CRO
group_1986-group_1998-group_2014	0.22	0.155	7.61E-07	-0.0673	0.0135	0.152	TET
group_1570-group_1673-group_668	0.0133	0.0188	9.18E-07	0.0905	0.0183	0.151	CIP
group_1821-group_1622-group_1145	0.018	2.29E-24	9.75E-07	0.118	0.024	0.15	TET
pilS1-pilS1_pilE_pilE_1_pilE1_pilS6_2_pilS5_pilS2_pilS6_pilS6_1_pilE_2_pilE_3-pilE_1_pilS6_pilE_pilE1	0.0104	0.324	9.80E-07	0.331	0.0673	0.15	PEN
group_1329-group_1882-group_1779	0.0342	6.56E-27	1.02E-06	0.0822	0.0167	0.15	TET
group_945-group_1522-group_816	0.952	2.05E-07	1.07E-06	0.301	0.0613	0.15	PEN
coaD-group_1369-group_1909	0.0493	3.17E-08	1.14E-06	0.102	0.0208	0.149	AZM
group_1514-group_679-group_464	0.763	3.19E-12	1.15E-06	0.157	0.032	0.149	PEN
group_447-group_1761-group_1801	0.0674	4.34E-08	1.20E-06	0.0616	0.0126	0.149	TET
group_484-group_956-group_829	0.0503	3.22E-10	1.41E-06	-0.0827	0.017	0.148	TET
rpoD-group_528-group_1522	0.0446	8.89E-10	1.54E-06	-0.402	0.0831	0.147	PEN
group_816-group_1522-group_528	0.0446	8.89E-10	1.54E-06	-0.402	0.0831	0.147	PEN
tbpA-group_1552-group_1864	0.0417	1.51E-08	1.62E-06	0.111	0.0231	0.147	AZM
group_494-group_1864-group_1552	0.0417	1.51E-08	1.62E-06	0.111	0.0231	0.147	AZM
group_947-group_1749-group_1801	0.0835	5.84E-07	1.70E-06	-0.059	0.0123	0.147	TET
group_1596-group_1720-group_75	0.0427	3.75E-55	1.89E-06	-0.11	0.0229	0.146	SMX
group_393-group_262-group_1450	0.0152	3.16E-05	1.94E-06	-0.104	0.0217	0.146	TET
group_171-group_1672-group_1229	0.107	3.42E-27	2.43E-06	0.228	0.048	0.145	CRO
group_1126-mafB_mafB_2-group_1187	0.756	4.03E-17	2.46E-06	0.131	0.0277	0.145	PEN

Table S3: Significant sv-pan-GWAS results for antibiotic resistance in the EuroGASP collection

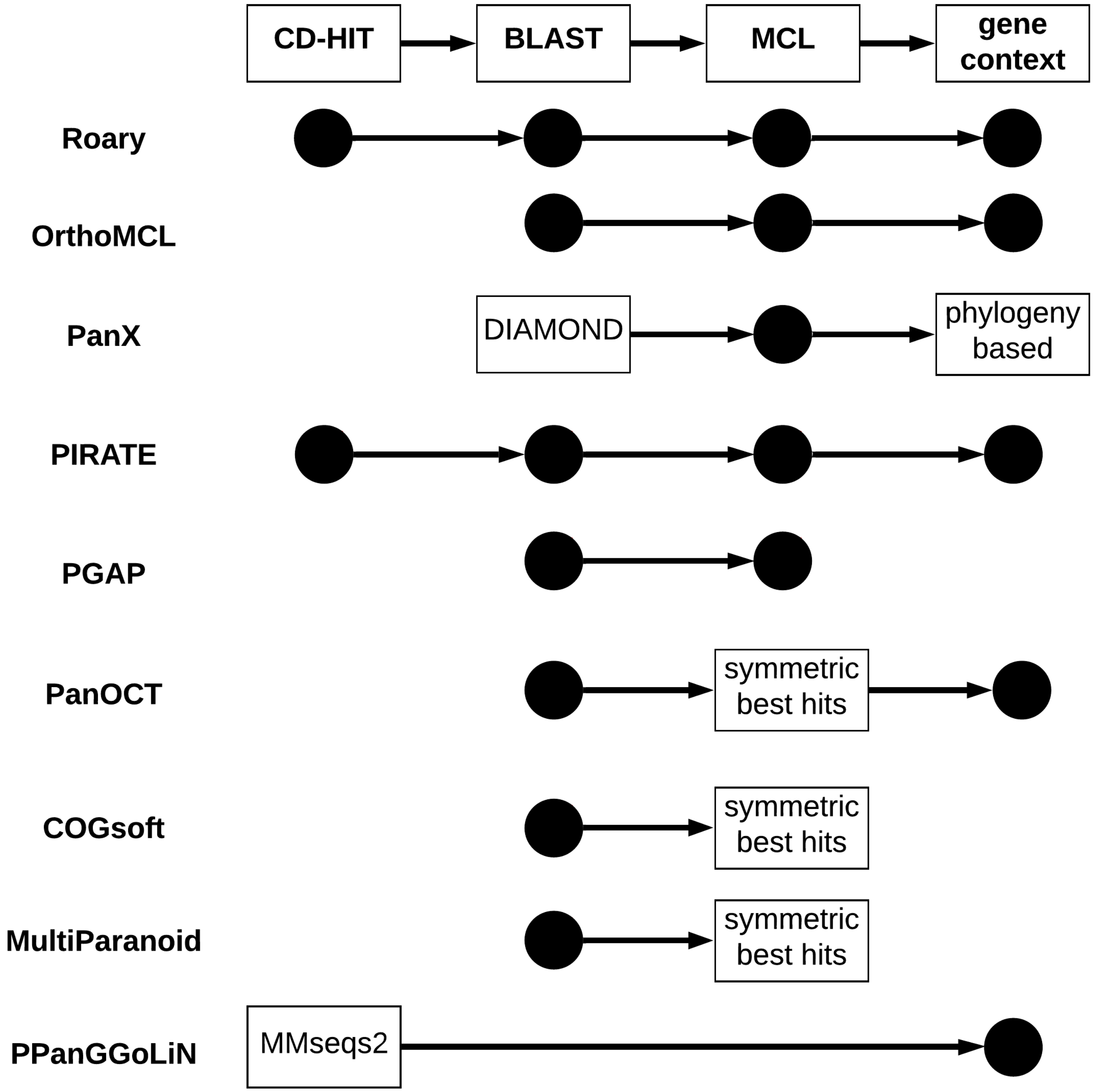

Figure S1: A comparison of the pipelines used by different pangenome analysis tools.

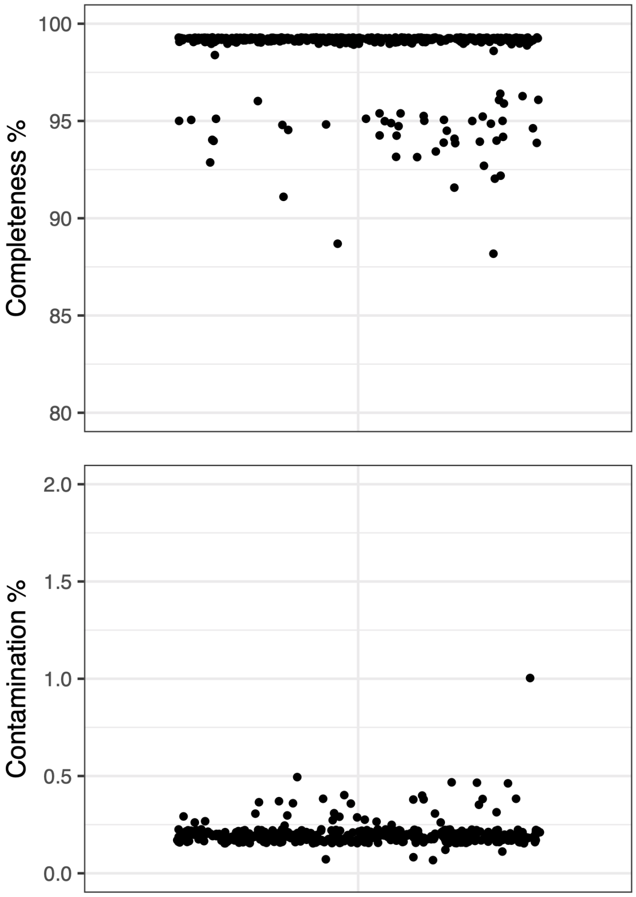

Figure S2: CheckM completeness and contamination estimates for the 413 *Mycobacterium tuberculosis* assemblies from an outbreak in London.

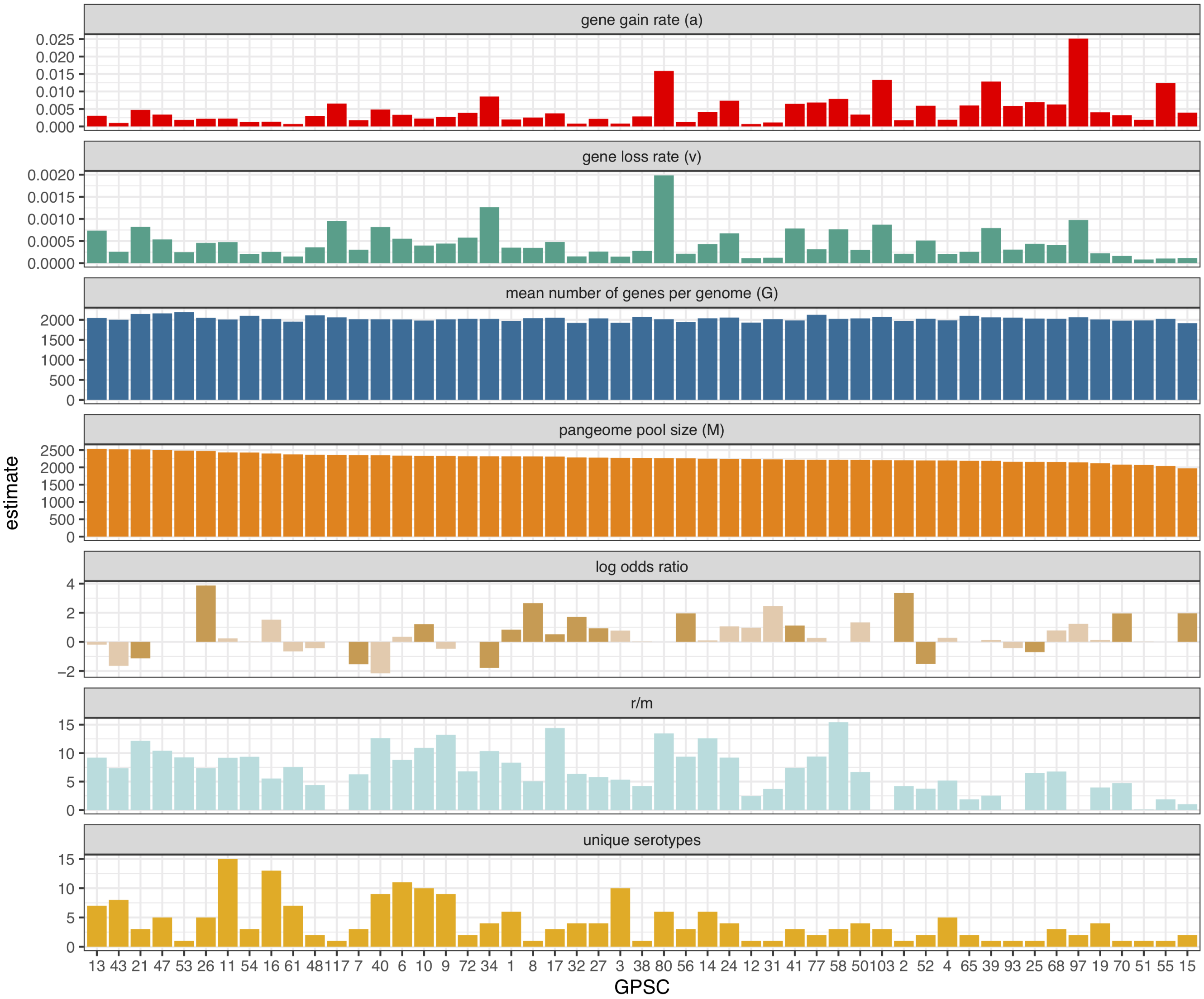

Figure S3: Inferred parameters for the Finitely Many Genes model for each of the 51 clusters of the global pneumococcal sequencing project for which reliable dated phylogenies could be inferred. The log odds ratio of invasive disease, number of unique serotypes and recombination rate given in (23) are also plotted for each cluster.

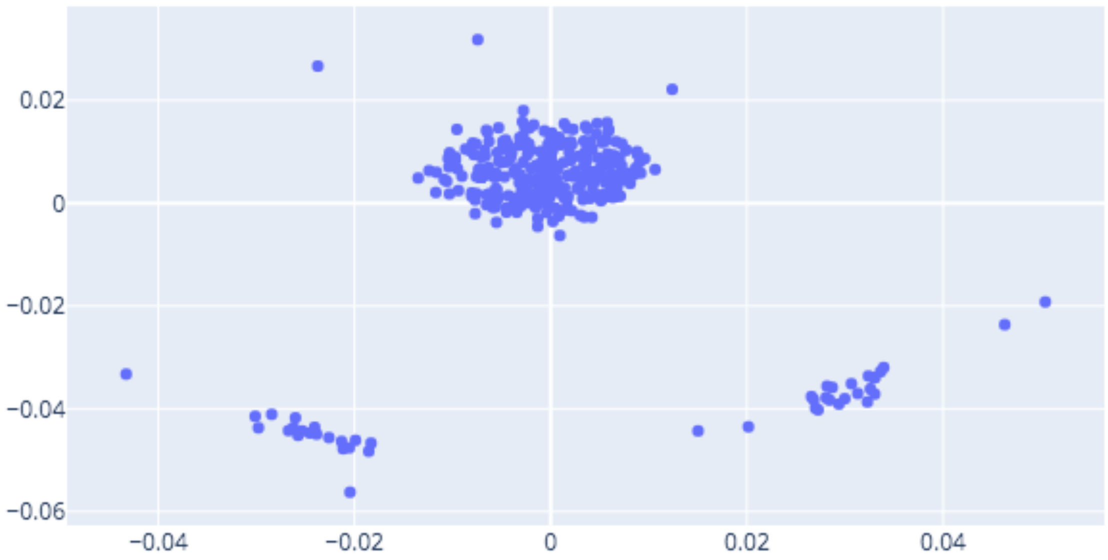

Figure S4: Multi Dimensional Scaling (MDS) plot of pairwise mash distances between isolates in the global *K. penumoniae* dataset. This plot is produced by the Panaroo quality control script.

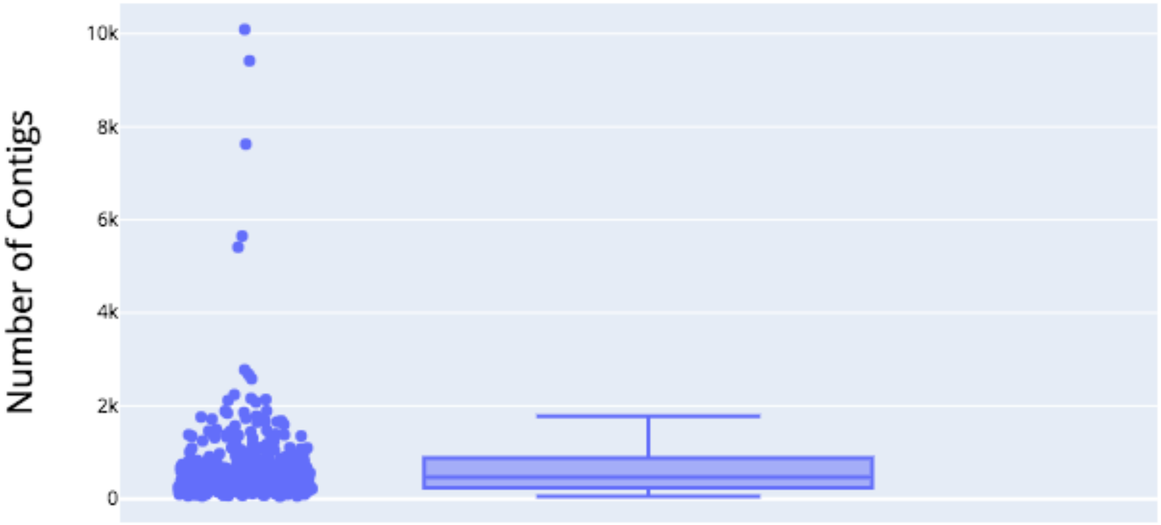

Figure S5: Boxplot produced by the Panaroo quality control script indicating the number of contigs in each of the *K. pneumoniae* assemblies.

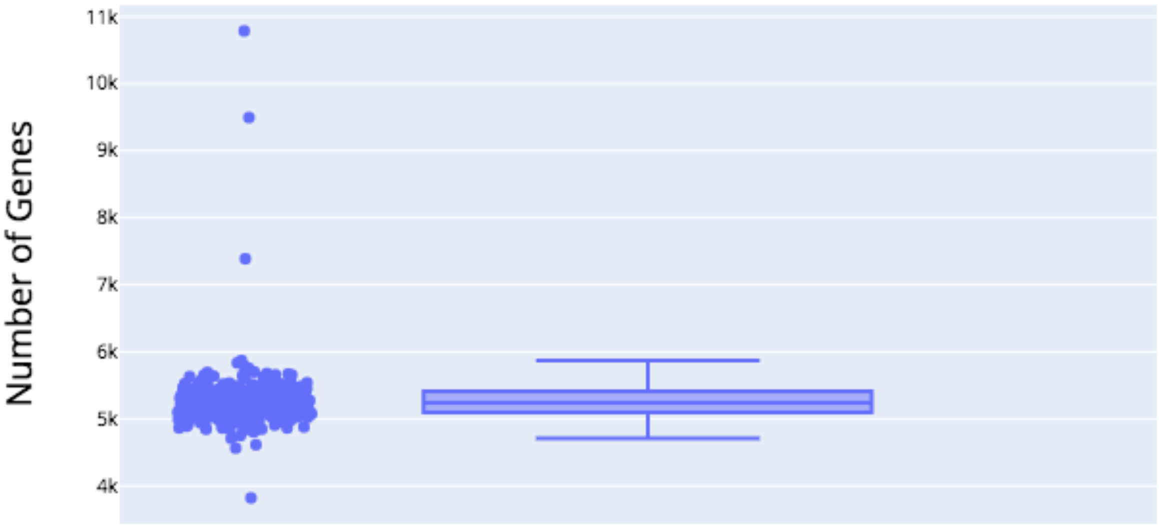

Figure S6: Boxplot produced by the Panaroo quality control script indicating the number of gene annotations in each of the *K. pneumoniae* assemblies.
